# Shear-Calibrated High-Intensity Interval Training to Promote Endothelial Autophagy and Delay Vascular Senescence: A Biomarker-Guided Approach

**DOI:** 10.3390/ijms27062653

**Published:** 2026-03-13

**Authors:** Amelia Tero-Vescan, Ylenia Pastorello, Mark Slevin

**Affiliations:** 1 Biochemistry Department, Faculty of Medicine in English, George Emil Palade University of Medicine, Pharmacy, Science, and Technology of Târgu Mureș, 540142 Târgu Mureș, Romania; amelia.tero-vescan@umfst.ro; 2 Department of Anatomy and Embryology, George Emil Palade University of Medicine, Pharmacy, Science, and Technology of Târgu Mureş, 540142 Târgu Mureş, Romania; 3 Center for Advanced Medical and Pharmaceutical Research, George Emil Palade University of Medicine, Pharmacy, Science, and Technology of Târgu Mureș, 540142 Târgu Mureș, Romania; mark.slevin@umfst.ro

**Keywords:** high-intensity interval training (HIIT), endothelial shear stress, autophagy, vascular ageing, senescence, AMPK–SIRT1–PGC-1α signalling, biomarkers

## Abstract

Vascular ageing is a complex process marked by progressive endothelial dysfunction, chronic low-grade inflammation (“inflammageing”), and reduced regenerative capacity, driven in part by an imbalance between protective endothelial autophagy and cellular senescence characterized by a proinflammatory senescence-associated secretory phenotype (SASP). Disruption of this autophagy–senescence axis accelerates vascular inflammation, arterial stiffening, and atherogenesis. High-intensity interval training (HIIT), consisting of repeated bouts of near-maximal anaerobic effort with recovery periods, is widely used by both elite and recreational athletes and is increasingly recognized as an effective nonpharmacological strategy to enhance endothelial function, arterial elasticity, and mitochondrial biogenesis. However, excessively intense or poorly structured HIIT, particularly in the absence of adequate recovery or in individuals with underlying cardiometabolic or vascular vulnerability, may induce endothelial stress and promote maladaptive vascular remodelling, including calcification and plaque instability. These considerations underscore the need for refined individualized exercise prescription strategies that balance performance benefits with endothelial protection. Based on these observations, here, we introduce a novel conceptual framework, “shear dose–calibrated HIIT,” designed to understand and define an optimal shear dose capable of maximizing autophagic flux while minimizing SASP activation. Experimental and clinical evidence of HIIT-induced effects on flow-mediated dilation (FMD), pulse wave velocity (PWV), and redox biomarkers is presented, followed by the proposal of a biomarker panel for assessing autophagic flux and cellular senescence in peripheral samples (peripheral blood mononuclear cells (PBMCs), extracellular vehicles (EVs), and plasma). This integrative approach, which combines vascular mechanotransduction, redox biology, and autophagic signalling, provides a novel translational perspective on how individually calibrated HIIT can promote vascular longevity and reduce cardiometabolic risk associated with aging and metabolic syndrome.

## 1. Introduction

The World Heart Report 2023 of the World Heart Federation estimates that cardiovascular disease (CVD) was the leading cause of death globally, accounting for 18.6 million deaths worldwide in 2019 (≈32% of all deaths worldwide, 9.6 million among males and 8.9 million among females), representing a substantial increase from 12.1 million deaths in 1990, when mortality was evenly distributed between sexes [1].

Comprehensive meta-analyses, such as the one conducted by Lang et al., encompassing over 20.9 million observations from 199 cohort studies, demonstrated a consistent inverse dose–response relationship between cardiorespiratory fitness (CRF) and the risk of disease and mortality [2]. Specifically, each 1 metabolic equivalent of task (MET) increase in CRF was associated with an approximately 18% lower risk of heart failure. In parallel, a meta-analysis of 85 studies reported that increasingly active individuals presented an ~20–40% lower risk of all-cause mortality and an ~30–40% lower risk of cardiovascular (CV) mortality [3]. A further analysis of an additional 20 studies involving 381,303 participants also demonstrated that individuals with higher estimated pulse wave velocity (PWV) (indicating higher shear stress impact), had significantly increased risks of total CV events, CV mortality, and all-cause mortality [4].

HIIT is an exercise modality characterized by repeated bouts of short-duration, high-intensity efforts performed near maximal aerobic capacity, interspersed with periods of active or passive recovery. Originally developed in athletes to improve aerobic power and performance, HIIT produces rapid fluctuations in cardiac output, blood flow velocity, and vascular shear stress, which stimulate endothelial nitric oxide production, mitochondrial biogenesis, and metabolic remodelling, making it an efficient stimulus for improving cardiorespiratory fitness, endothelial function, and vascular elasticity.

Early experimental evidence demonstrated that aerobic interval training performed at 90–95% of maximal heart rate significantly improved maximal oxygen uptake (VO_2_max) compared with moderate continuous training performed at equivalent total work. These improvements were largely attributed to increases in stroke volume and enhanced cardiovascular efficiency, highlighting the strong physiological stimulus generated by HIIT [5]. Across different age groups and disease states, HIIT, compared with moderate-intensity continuous training, consistently enhances cardiorespiratory fitness and promotes vascular adaptations associated with improved cardiovascular health. These benefits include clinically meaningful improvements in endothelial function, commonly assessed by FMD, which reflects increased nitric oxide bioavailability and endothelial responsiveness to hemodynamic stimuli. In addition to improving endothelial function, exercise training also reduces arterial stiffness, a key determinant of vascular ageing and cardiovascular risk. Arterial stiffness is most frequently evaluated by PWV, where lower PWV values indicate improved arterial elasticity. Consistent with these mechanisms, a meta-analysis of 22 studies including 619 participants demonstrated that HIIT significantly improves FMD and reduces PWV, highlighting its beneficial effects on vascular function and arterial compliance [6]. However, responsiveness is heterogeneous: females show significantly greater increases in cerebral blood velocity than males do, and while some individuals show large improvements, others plateau or even display transient adverse vascular signals [7]. For example, Paravlic and Drole reported that FMD measured in the brachial artery varied significantly depending on a person’s existing CVD risk factors [8], whereas blood pressure changes also notably varied in HIIT studies indicative of responder and nonresponder groups [9], and these multifactorial ‘risk parameters’ will be discussed in more detail later in this review.

A key reason is that most HIIT prescriptions (noting that the majority of individuals have had no analysis of their HIIT for too long) are defined by external workload (percent of peak power/speed or heart rate zones) rather than arterial sheer stress, which is the direct proximal endothelial stimulus. Arterial shear is the frictional force exerted by flowing blood on the endothelial surface. It orchestrates nitric oxide (NO) bioavailability, Ras homologue family member A (rhoA)–Rho-associated coiled-coil-containing protein kinase (ROCK) and nuclear factor kappa-light-chain-enhancer of activated B cells (NF-κB) tone, and the AMPK–SIRT1–PGC 1α axis, which controls mitochondrial biogenesis and redox homeostasis [10]. Shear also interfaces with autophagy and mitophagy, the housekeeping programmeme that removes damaged organelles, and with cellular senescence, a stress response that confers a critical proinflammatory secretome (SASP) [11]. In recent years, evidence has demonstrated that the autophagy–senescence pathways are master modulators and determinants of healthy ageing, whereas their deregulation closely corresponds to chronic and pathological disease progression, such as Alzheimer’s disease (AD) and CVD, and ultimately mortality risk [12].

We provide strong evidence that the shape/profile and dose of shear generated by HIIT, set by bout intensity, duration, cadence, and recovery, when stratified according to a specific individual’s clinical and genetic profile, determine whether endothelial cells enter a rejuvenating state (autophagy ↑, SASP ↓) or a maladaptive state (microparticles ↑, SASP ↑).

In this context, the present review advances a practical concept: shear-calibrated HIIT. Instead of one-for-all identical intervals, we advocate prescription work and recovery to elicit an individualized shear band that maximizes autophagic flux while suppressing senescence, thereby mediating superior changes in FMD and PWV and supporting glycocalyx integrity [13]. We provide a concise signal transduction map linking shear stress to autophagy/mitophagy and SASP signalling and propose a biomarker toolbox with recommended sampling windows as part of a minimal, clinically applicable protocol for estimating and targeting shear stress. This article is structured as a narrative review that integrates current experimental and clinical evidence to propose a mechanistic framework for shear-calibrated HIIT and its potential role in promoting endothelial autophagy and delaying vascular ageing. Through this integrative perspective, we aim to highlight how individualized shear-dose calibration may help minimize the long-term risk of maladaptive cardiovascular remodelling, as eloquently discussed in the 2026 systematic review of Saulicz et al. [14].

## 2. High-Intensity Interval Training and the Redox–Autophagy Cycle: Mitophagy Activation and SASP Suppression in Endothelial Ageing

Over time, repeated excessive mechanical, oxidative, and metabolic stresses induce alterations in ECs, transforming their NO-producing phenotype into a proinflammatory and vasoconstrictive phenotype characterized by impaired autophagy, mitochondrial dysfunction, and progressive cellular senescence [15]. A study in primary human aortic (HAEC) and coronary artery (CAEC) endothelial cells subjected to replicative senescence (passages P1–P12) revealed that endothelial nitric oxide synthase (eNOS) expression and NO bioavailability declined progressively, while the di/tetrahydrobiopterin (BH_2_/BH_4_) ratio increased significantly, indicating that eNOS uncoupling and the levels of markers of oxidative/nitrosative stress and inflammation (e.g., superoxide, peroxynitrite, cyclophilin A, EMMPRIN, and matrix metalloproteinase (MMP)-9/13) were elevated, collectively linking cellular senescence with endothelial dysfunction and proinflammatory pathways [16].

In particular, under conditions of disturbed or oscillatory flow, endothelial cells accumulate depolarized mitochondria and reduce AMPK activity while increasing the expression of SASP factors such as interleukin-6 (IL-6), tumour necrosis factor-alpha (TNF-α), plasminogen activator inhibitor-1 (PAI-1), and growth differentiation factor 15 (GDF15; a SASP-linked stromal stress signal induced by mitochondrial dysfunction) [11]. The resulting proinflammatory milieu amplifies vascular inflammation, matrix remodelling, and arterial stiffening, which are key hallmarks of vascular ageing. Using in vitro shear stress systems in HAECs and umbilical vein ECs combined with disturbed-flow EC-specific uncoupling protein 2 (UCP2) knockout mice, it was reported that in vitro, unidirectional shear stress, as observed in HIIT, induced Krüppel-like factor 2 (KLF2)-dependent upregulation of UCP2, whereas oscillatory shear suppressed UCP2 expression. In vivo, endothelial UCP2 deficiency promoted vascular inflammation, collagen deposition, and carotid artery atherosclerotic plaque formation through inactivation of AMPK and promotion of forkhead box O transcription factor 1 (FOXO1) proinflammatory stimulation [17].

In db/db and C57BL/6 mouse aortas subjected to high-glucose/high-lipid conditions, exogenous GDF15 administration (acute or prolonged) significantly improved endothelium-dependent relaxation and reduced endothelial reactive oxygen species (ROS) production while normalizing NADPH oxidase 2 (NOX2), NRF2, angiotensin-converting enzyme (ACE), and ACE2 expression. This process operates through an AMPK-dependent mechanism, as evidenced by increased AMPK phosphorylation and the abrogation of GDF15′s vasoprotective effects by the AMPK inhibitor Compound C. A partial mechanism for this mechanism was suggested in a review by Khalafi et al. [18], which elegantly described how HIIT produces pulsatile surges of hemodynamic shear stress that act as primary mechanotransductive cues that recalibrate endothelial metabolism and redox signalling. They reported how each transient shear episode evoked a short-lived increase in ROS, which acts as a hormetic signal to activate the redox–autophagy axis, particularly AMPK, SIRT1, and PGC-1α, which together are able to coordinate energy sensing, mitochondrial quality control, and antioxidant gene expression [10,18]. Through this integrated signalling cascade, exercise-induced shear promotes mitophagy, enhances mitochondrial biogenesis, and restores NO bioavailability, maintaining vascular homeostasis even under metabolic stress.

Recent work has provided a proof-of-concept for physical exercise as a physiological senomorphic intervention capable of attenuating senescence-associated signalling without the need to utilize potentially cytotoxic pharmacologic senolytics such as dasatinib, quercetin, fisetin and the senomorphics metformin, rapamycin or NAD^+^ precursors, which have demonstrated efficacy in reducing the SASP and clearing senescent cells in preclinical and early-phase clinical studies (for a review see [15]). Exercise-induced hemodynamic shear stress may therefore represent a readily accessible nonpharmacological strategy to sustain autophagic flux and suppress SASP signalling, slowing the process of endothelial dysfunction and arterial ageing [19].

For example, in aged mice (18 months old) subjected to structured exercise training, analysis of endothelial mitophagy revealed that exercise increased PPARγ-dependent FUNDC1 mitophagic flux in cardiac microvascular endothelial cells, concomitantly reduced the expression of an endothelial senescence marker (β-galactosidase), and improved coronary microvascular function. Endothelial FUNDC1 deficiency abolished these protective effects and exacerbated ischemia–reperfusion injury, demonstrating that exercise-induced shear preserved vascular homeostasis through mitophagy-mediated mitochondrial quality control and that the modulation of mitophagy was sufficient to protect the endothelium [20].

In addition to its classical role in NO production, regular exercise regulates redox–autophagy cycling, which is a dynamic interface that integrates metabolic and mechanical stress responses. Properly calibrated HIIT protocols (by interval intensity, cadence, and recovery duration) should optimize the shear dose to maximize mitophagy turnover while minimizing prosenescent signalling. Such adaptations have led to improved FMD, reduced PWV, and preservation of the endothelial glycocalyx, which is a critical and key marker of vascular youthfulness (as described in a paper “is it possible to train the endothelium?” [21]). In female Sprague–Dawley rats assigned to young sedentary (8-month-old), old sedentary (26-month-old), moderate-intensity continuous training (MICT), or long-term HIIT groups, a chronic 8-month exercise intervention revealed that, compared with MICT, HIIT robustly activated AMPK signalling, significantly increased mitochondrial biogenesis and mitophagy markers, and enhanced antioxidant capacity (superoxide dismutase 2 (SOD2)) in these animals. HIIT alone also promoted mitochondrial supercomplex assembly in ageing soleus muscles, thereby partially restoring mitochondrial function and muscle function impaired by ageing [22].

Taken together, these observations suggest that HIIT could be a potential precision mechanotherapeutic tool that, by supporting the restoration of mitochondrial homeostasis, could help maintain endothelial regenerative capacity and inhibit the chronic inflammatory feedback loops that drive vascular ageing. The following sections present in detail the molecular signalling pathways underlying this adaptation, including AMPK–SIRT1–PGC-1α/NRF2 activation, PTEN-induced kinase 1 (PINK1)/Parkin-mediated mitophagy, and SASP inhibition, to elucidate how mechanical forces are transduced into sustained molecular resilience within the vascular endothelium.

### 2.1. Endothelial Metabolic and Mechanical Coupling Through the AMPK–SIRT1–PGC-1α/NRF2 Pathway

Under physiological laminar shear stress, ECs experience transient changes in their energetic state that increase the AMP/ATP ratio, leading to the activation of AMPK, a master regulator of cellular metabolism and autophagy [23]. Activated AMPK phosphorylates unc-51-like autophagy-activating kinase 1 (ULK1) at Ser317 and Ser777, initiating the early stages of autophagosome formation and suppressing mechanistic target of rapamycin complex 1 (mTORC1) signalling to favour catabolic flux (reviewed by [24]). In parallel, AMPK enhances NAD^+^ bioavailability, thereby stimulating the deacetylase SIRT1, which targets the key transcriptional regulators PGC-1α and FOXO3a. SIRT1-mediated deacetylation of these proteins increases the transcription of genes involved in mitochondrial biogenesis, antioxidant defence, and metabolic flexibility [25].

The coactivator PGC-1α functions as a central hub linking energy metabolism and redox homeostasis. Once activated, PGC-1α cooperates with NRF2 to promote the expression of the canonical antioxidant enzymes SOD2, catalase, and heme oxygenase-1 (HO-1), which detoxify mitochondrial ROS. NRF2 also induces genes involved in mitochondrial quality control and mitophagy, such as p62/SQSTM1 and NDP52, which integrate antioxidant and autophagic defences [26,27].

Using in vitro flow systems and multiscale imaging of whole arteries from ApoE^−/−^ mice, Nasr et al. demonstrated that low shear stress (LSS), but not HSS, activated a VPS34-independent, PI3KCIIα-dependent autophagy program in ECs. Partial genetic loss of PI3KCIIα (ApoE^−/−^PI3KCIIα^+/−^ mice) impaired endothelial autophagic flux, enhanced mTORC1 signalling, reduced primary cilium formation, and resulted in marked endothelial dysfunction and increased atherosclerotic plaque burden, specifically in LSS-exposed arterial regions. These deficiencies were selectively rescued by rapamycin-mediated mTORC1 inhibition [28]. Male C57BL/6 mice that were subjected to alternate-day fasting or time-restricted fasting combined with treadmill running for 6 weeks presented significantly improved glucose homeostasis (increased muscle glycogen and reduced glycated serum proteins). Molecular analyses by Western blotting revealed significant activation of the AMPK–SIRT1–PGC-1α axis with upregulation of glucose transporter 4 (GLUT4), peroxisome proliferator-activated receptor gamma (PPAR-γ), and NRF2/HO-1 signalling and suppression of FoxO1. Notably, time-restricted fasting plus exercise elicited stronger metabolic and antioxidant responses than alternate-day fasting did. These findings support the concept of a positive synergistic effect of moderate exercise and fasting on systemic metabolic regulation [29].

By investigating the mechanism in greater depth, we find that the AMPK–SIRT1–PGC-1α/NRF2 axis forms a redox–autophagy circuit that enables ECs to sense, detect and resolve oxidative stress. Transient ROS bursts produced during shear stress or exercise can act as hormetic stimuli that briefly increase oxidative signalling but subsequently trigger AMPK-dependent autophagy and NRF2-driven antioxidant gene expression, restoring redox equilibrium and preserving eNOS activity [30]. These beneficial effects were shown via an experimental model of hypertension in which six-week-old male spontaneously hypertensive (SHR) rats were subjected to 12 weeks of high-intensity treadmill training (70–80% VO_2_max). Compared with sedentary SHR controls, exercise-treated SHRs exhibited significantly improved endothelium-dependent aortic relaxation, increased eNOS Ser1177 phosphorylation, reduced oxidative stress (↓ NOX4, ↓ DHE and 8-OHdG staining), and marked suppression of NLRP3 inflammasome-driven pyroptosis, and these effects were mechanistically associated with robust activation of the AMPKα–SIRT1 signalling axis [31].

**Figure 1:**
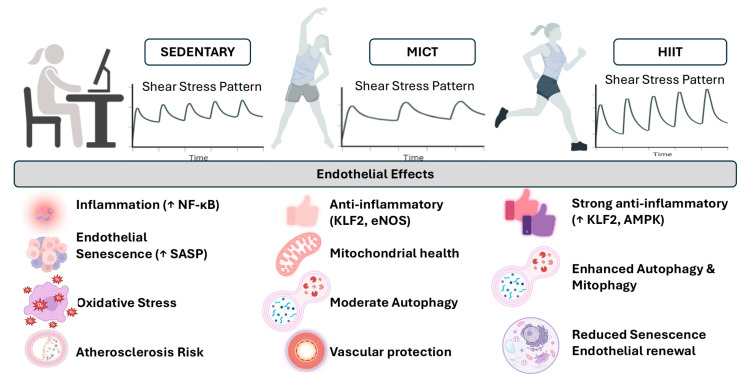
Hemodynamic shear stress patterns induced by sedentary behaviour, MICT, and HIIT, and their endothelial consequences. Sedentary conditions are characterized by low or disturbed shear stress, which promotes endothelial dysfunction, inflammation, oxidative stress, and SASP signalling, thereby accelerating vascular ageing and atherogenesis. MICT induces moderate laminar shear stress that supports endothelial homeostasis through improved eNOS activity, mitochondrial function, and modest stimulation of autophagy. In contrast, HIIT generates repeated high-magnitude pulsatile laminar shear stress that strongly activates mechanosensitive pathways, including AMPK–SIRT1–PGC-1α/NRF2 signalling, autophagy, and antioxidant defences. When appropriately calibrated, this response improves endothelial function (↑ FMD), reduces arterial stiffness (↓ PWV), and preserves glycocalyx integrity. However, excessive or poorly calibrated shear may transiently increase vascular stress in individuals with advanced vascular disease, highlighting the need for individualized shear-dose calibration. (NF-κB, nuclear factor kappa-light-chain-enhancer of activated B cells; HIIT, high-intensity interval training; MICT, moderate-intensity continuous training; SASP, senescence-associated secretory phenotype; eNOS, endothelial nitric oxide synthase; FMD, flow-mediated dilation; AMPK, adenosine monophosphate-activated protein kinase; SIRT1, sirtuin 1; PGC-1α, peroxisome proliferator-activated receptor-gamma coactivator-1α; NRF2, nuclear factor erythroid-2-related factor 2; PWV, pulse wave velocity, ↑—up arrow means increase.) Created in BioRender. Tero-Vescan, A. (2026) https://BioRender.com/83o2n51 (accessed on 5 March 2026).

Conversely, disturbed or oscillatory flow suppresses AMPK signalling and reduces SIRT1 activity, leading to mitochondrial depolarization, impaired autophagy, and the accumulation of dysfunctional mitochondria that overproduce ROS [32]. These alterations amplify SASP mediators, notably IL-6, TNF-α, and PAI-1, or amplifiers, such as monomeric C-reactive protein, that drive endothelial inflammation and matrix remodelling and accelerate vascular ageing [33]. Both low shear stress and oscillatory flow impaired endothelial barrier function in vitro (HUVEC) and in vivo (ApoE^−/−^ mice) via an autophagic flux-dependent mechanism. This is extremely important to recognize in the context of defining safe HIIT prescriptions, as described in the figure and legend below. The hemodynamic shear stress patterns induced by sedentary behaviour, MICT, and HIIT and their associated endothelial consequences are presented in Figure 1.

**Figure 2:**
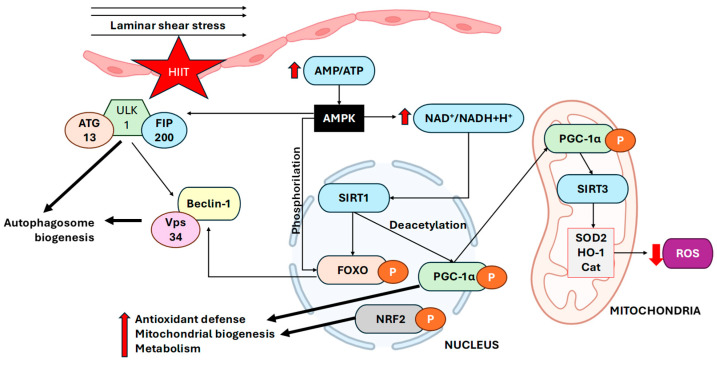
Integration of metabolic and mechanical signals in ECs: AMPK–SIRT1–PGC-1α/NRF2-mediated redox control and ULK1–Beclin-1–LC3-driven autophagy under shear stress. HIIT increases laminar shear stress and transiently elevates the AMP/ATP ratio, activating AMPK. AMPK phosphorylates and activates ULK1 and FIP200, promoting autophagy initiation through class III PI3K/Beclin-1/VPS34 complex. Concurrently, AMPK increases the NAD^+^/NADH ratio, leading to the activation of SIRT1 and SIRT3. SIRT1 deacetylates FOXO and PGC-1α, promoting the transcription of antioxidant and mitochondrial biogenesis genes in cooperation with NRF2. Within mitochondria, PGC-1α and SIRT3 further activate antioxidant enzymes, including SOD2, HO-1, and Cat, reducing ROS accumulation. Overall, this adaptive response enhances mitochondrial turnover, redox homeostasis, and endothelial metabolic resilience during HIIT. (HIIT—high-intensity interval training, AMPK—AMP-activated protein kinase, AMP/ATP—adenosine monophosphate/adenosine triphosphate, NAD^+^/NADH+H^+^—nicotinamide adenine dinucleotide (oxidized/reduced), SIRT1,3—sirtuin 1,3, PGC-1α—peroxisome proliferator-activated receptor gamma coactivator 1-alpha, FOXO—forkhead box O, NRF2—nuclear factor erythroid 2-related factor 2, ULK1—unc-51-like kinase 1, FIP200—FAK family-interacting protein of 200 kDa, VPS34—vacuolar protein sorting 34 (class III PI3K), ATG13—autophagy-related protein 13, SOD2—superoxide dismutase 2, HO-1—heme oxygenase-1, Cat—catalase, ROS—reactive oxygen species. Down red arrow means decreased effect, up red arrow means increased effect.) Created in BioRender. Tero-Vescan, A. (2026) https://BioRender.com/pj147fe (accessed on 5 March 2026).

Therefore, in healthy individuals, HIIT reactivates this protective AMPK–SIRT1–PGC-1α/NRF2 pathway through cyclic hemodynamic shear stress. Controlled studies have demonstrated that repeated bouts of HIIT increase AMPK phosphorylation and SIRT1 expression in human and rodent endothelium while concurrently enhancing PGC-1α and NRF2 signalling, thereby increasing antioxidant enzyme levels and restoring autophagic flux; see Figure 2 [34]. For example, moderately trained young men (mean age 25 years) who underwent an 8-week moderate cycling intervention with interspersed 30 s sprints presented acute post-exercise activation of the AMPK–ULK1 axis (↑ AMPK Thr172 and ULK1 Ser317 phosphorylation) without mTORC1 activation, followed by increased LC3-I/II and BNIP3 protein content during early recovery, in skeletal muscle biopsies. Interestingly, chronic training alone increased the basal expression of oxidative phosphorylation (OXPHOS) complex I, Parkin, and BNIP3, indicating a partial increase in autophagy and mitophagy regulatory capacity independent of sprint inclusion [35].

Similarly, male Wistar rats that underwent 5 × 5 cycles of HIIT treadmill running presented broader oxidative remodelling and post-training gene and protein upregulation of the AMPK–PGC-1α network and mitochondrial/antioxidant markers (Ampkα1, PGC-1α, and Nrf1/2) in the soleus muscle (slow-twitch fatigue resistant) than did the extensor digitorum longus (fast-twitch glycolytic) in the middle exercising controls. The data strongly supported a muscle-typology-dependent AMPK-linked oxidative adaptation that was more pronounced with HIIT than with MICT [36]. Upregulation of the AMPK signalling cascade was also observed in rodent models of myocardial ischemia–reperfusion injury following 4–12 weeks of treadmill-based HIIT. Compared with sedentary controls, downstream signalling again includes the activation of PGC-1α–dependent mitochondrial biogenesis and the enhancement of LC3II/PINK1/Parkin-mediated mitophagy, thereby improving mitochondrial quality control, reducing ROS accumulation, limiting mitochondrial permeability transition pore opening, and ultimately decreasing infarct size by approximately 30–40% while improving postischemic cardiac functional recovery [37].

Collectively, these findings identify the AMPK–SIRT1–PGC-1α/NRF2 axis as a central signalling hub linking mechanical and metabolic stimuli to mitochondrial homeostasis, autophagy, and redox balance. Through this mechanism, HIIT functions as a physiological senomorphic intervention, restraining SASP activity while promoting mitochondrial renewal and endothelial longevity.

### 2.2. Regulation of Endothelial Autophagic Flux Through the ULK1–Beclin-1–LC3 Axis Under Shear Stress

In ECs, autophagic flux represents the efficiency of cellular self-renewal under mechanical and metabolic stress, acting as a critical determinant of vascular homeostasis versus senescence. Downstream of AMPK activation and mTORC1 inhibition, the ULK1 complex (ULK1–ATG13–FIP200) initiates autophagosome biogenesis and licences the class III PI3K known as VPS34/Beclin-1 machinery, whereas the ATG5–ATG12/ATG16L1 complex extends the isolation membrane; during elongation, cytosolic LC3-I is lipidated to LC3-II and incorporated into autophagosomal membranes, enabling cargo sequestration and flux to lysosomes [38]. Autophagy guidelines emphasize that LC3-II together with p62/SQSTM1 turnover (±lysosomal blockade) is the appropriate readout of autophagic flux, not static LC3 levels [39].

A study in HUVECs showed that vascular endothelial growth factor (VEGF) transiently induced autophagy via AMPKα1-dependent phosphorylation of ULK1 at Ser556, followed by phosphorylation of downstream initiation components (ATG14 Ser29, ATG16L1 Ser278) and increased LC3B-II formation, with increased autophagic flux. This proautophagic signal was subsequently inhibited by delayed mTOR activation [40]. Under normal physiological conditions, VEGF expression is mechanoprotective and promotes autophagy and flux, particularly in association with laminar shear stress and in response to vessel hypoxia/HIF-1α expression; however, during abnormal oscillatory disturbed flow, VEGF is maladaptively uncoupled from eNOS and activates mTORC1 while suppressing AMPK and ULK1, blocking autophagic flux and potentially leading to both pathological angiogenesis and a build-up of toxins/senescence damaging the mitochondria and causing vascular injury [41,42,43]. This indicates the duality of the critical signalling pathways and hence the necessity to understand and stratify response to exercise patterns on an individual basis. The presence of specific chronic diseases or comorbidities provides additional evidence, as described below.

Physiologic shear involves different endothelial autophagy circuits; for example, HUAECs under controlled flow conditions presented increased levels of the autophagy markers ULK1–Beclin-1 signalling and LC3-II, whereas arteries from ApoE^−/−^ mice, as assessed by en face LC3B imaging with chloroquine flux, presented selective loss of the LC3B signal and worsened dysfunction/plaque deposition in low shear stress (LSS) regions. This effect was also reversed by mTORC1 inhibition, which is consistent with a PI3KCIIα–mTORC1 axis that is designed to protect atheroprone low-shear territories. Shear-pattern-specific autophagy readouts were demonstrated in HUAECs exposed for 24 h to LSS (1 dyne/cm^2^) and high shear (HSS) (18 dynes/cm^2^), as LSS-driven autophagy required PI3KCIIα (not VPS34), whereas HSS required VPS34 (not PI3KCIIα) [28].

Therefore, in a perfectly designed physiologically balanced organism, HIIT provides repeated shear bouts that help maintain autophagic flux and mitochondrial health, limiting the accumulation of damaged proteins/organelles [21]. A systematic review and meta-analysis of 13 randomized controlled trials revealed that even in patients with type 2 diabetes mellitus (but no other comorbidities), exercise training significantly improved endothelial function, as assessed by brachial artery flow-mediated dilation, with HIIT producing the greatest effect, specifically when performed more than three times per week, in sessions shorter than 60 min, and for a total of ≤180 min per week, which indicates that overtraining beyond this duration negates any positive effects [44]; see Figure 2.

Moreover, the clinical susceptibility to vascular damage and disease, as we have previously stated, indicates that proper stratification of HIIT is important to ensure the avoidance of detrimental effects. For example, in preclinical studies, a systematic review and meta-analysis that included 23 experimental studies and 1033 mice found that high-fat diet-induced diabetes consistently impaired hepatic autophagy, as evidenced by the absence of p62 degradation, increased p-mTOR/mTOR signalling, and a reduced p-AMPK/AMPK ratio. Further decreases in ATG7 and LC3-II were observed when a high-fat diet was combined with streptozotocin, indicating a shift toward mTOR-driven autophagy inhibition that was exacerbated by hyperglycemia, prolonged high-fat diet exposure, and ageing [45].

Importantly, the efficiency of autophagic flux acts as a critical boundary condition: when autophagy remains sufficient, ROS and inflammatory signalling are effectively contained; however, when flux becomes inadequate, as occurs under disturbed shear stress, ECs release increased amounts of microparticles and extracellular vesicles that propagate vascular dysfunction [46]. Recent mechanobiology overviews identify disturbed shear stress-induced endothelial microparticle (EMP) release as a hallmark of endothelial injury (and therefore a potential biomarker), whereas steady shear stress, coupled with improved mitochondrial health, is consistently associated with reduced microparticle shedding and preserved endothelial integrity [47].

Together, these findings identify endothelial autophagic flux through the ULK1–Beclin-1–LC3 axis as a shear-sensitive, context-dependent quality-control system that integrates mechanical cues, VEGF signalling, and metabolic status to preserve mitochondrial integrity and vascular homeostasis. Physiological laminar or exercise-induced shear supports AMPK-biassed autophagy and efficient flux, whereas disturbed flow, mTORC1 dominance, and metabolic comorbidities impair flux, promote microparticle release, and accelerate endothelial dysfunction and vascular ageing, hence establishing the need for individualized stratification of exercise shear dose and disease context.

### 2.3. Mechanosensitive Regulation of the PINK1/Parkin Mitophagy Axis Defines the ‘Stability’ of the Vascular Endothelium

Mitophagy plays a major protective role by mitigating alterations induced by oxidative stress, such as inflammatory processes, glycocalyx degradation, and arterial stiffening [48]. Under disturbed or oscillatory flow, ECs accumulate depolarized mitochondria, downregulate AMPK activity, and upregulate SASP mediators, fuelling vascular inflammation, matrix remodelling, and arterial stiffening, which are the strongest indicators of vascular ageing [25].

**Figure 3:**
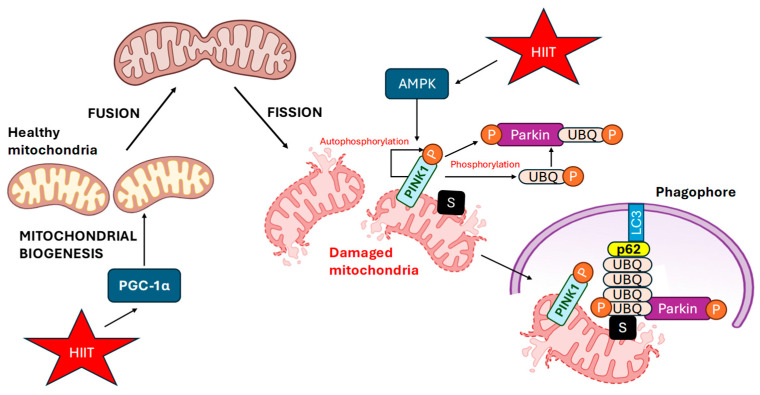
Controlled HIIT-induced activation of AMPK and PGC-1α promotes PINK1/Parkin-dependent mitophagy and mitochondrial quality control. In healthy mitochondria with a normal membrane potential, PTEN-induced kinase 1 (PINK1) is imported into the inner mitochondrial membrane and degraded. When mitochondria become depolarized and damaged, PINK1 accumulates on the OMM and undergoes autophosphorylation. PINK1 then phosphorylates both UBQ and Parkin, leading to Parkin activation. Activated Parkin ubiquitinates several outer S membrane proteins (VDAC1, Mfn1, Mfn2, TOM20, etc.), marking damaged mitochondria for degradation. Ubiquitinated substrates are recognized by the autophagy receptor p62, which interacts with LC3 on the forming phagophore, resulting in the engulfment of damaged mitochondria into an autophagosome for lysosomal degradation. HIIT stimulates AMPK-PINK1/Parkin-dependent mitophagy to eliminate damaged mitochondria and promote mitochondrial quality control. (PINK1, PTEN-induced kinase 1; Parkin, E3 ubiquitin ligase; UBQ, ubiquitin; P, phosphate group (phosphorylation); p62, sequestosome 1; LC3, microtubule-associated protein 1A/1B-light chain 3; S, proteins considered substrates located on the outer mitochondrial membrane ubiquitinated by Parkin; OMM, outer mitochondrial membrane; phagophore, membrane precursor that engulfs the damaged mitochondrion during autophagosome formation; HIIT, high-intensity interval training; AMPK, AMP-activated protein kinase.) Created in BioRender. Tero-Vescan, A. (2026) https://BioRender.com/invi4t2 (accessed on 5 March 2026).

In this context, HUVECs that were exposed for 24–48 h to disturbed flow via a clinostat-simulated microgravity model exhibited mitochondrial depolarization, Drp1-mediated fission and robust activation of PINK1/Parkin-dependent mitophagy, which limited mitochondrial ROS accumulation and suppressed NLRP3 inflammasome activation. Genetic or pharmacological inhibition of mitophagy (PINK1 siRNA or mdivi-1) exacerbates IL-1β release, MMP1 expression, endothelial hyperpermeability and cell migration, demonstrating that PINK1-driven mitophagy is able to preserve endothelial barrier integrity under mechanical unloading [49]. PINK1 was also demonstrated to be a good marker of mitochondrial stress, and its expression and translocation in normal cells distinguished it from those that are degraded and targeted for mitophagy in mouse embryonic fibroblasts and a variety of human cell lines. In the latter, PINK1 accumulates on the outer mitochondrial membrane, where it activates and recruits the E3 ubiquitin ligase Parkin, which is known to ubiquitinate surface proteins, marking the mitochondria for autophagic degradation [50]. For more information see Figure 3.

In terms of signalling pathways, Coon et al. used a whole-genome CRISPR screen in a mouse aortic endothelial Klf2:d2EGFP reporter line stimulated by laminar shear stress (18 dyn/cm^2^) to identify a mitochondrial/mitophagy arm in which mitochondrial Ca^2+^/ROS promoted PINK1-dependent mitophagy and p62 scaffolding to amplify MEKK2/3–MEK5–ERK5 signalling. Using Doppler and micro-CT, they reported that PINK1^−/−^ mice (C57BL/6J background) presented reduced ERK5 nuclear translocation and diminished endothelial Klf2 and eNOS expression in the thoracic aorta, alongside defective flow-driven vascular remodelling after femoral artery ligation [51]. A very recent study also revealed that chronic hypoxia resulted in reduced lung tissue expression of Parkin and PINK1, which was associated with decreased mitophagy during pulmonary hypertension, in a murine model. In addition, Parkin/PINK1 knockout models presented increased levels of hypertension associated with VSMC phenotypic switching and reduced mitophagy. These data strongly support a critical role for the Parkin/PINK pathway in the maintenance of vascular protection associated with shear or oscillatory stress [52].

In vivo exercise models provide physiological support for shear-dependent mitochondrial quality control, showing that mimicking endurance or interval training in mice enhances endothelial anti-inflammatory signalling and favourable vascular remodelling in high-flow arteries, with reduced mitochondrial but increased cytosolic PINK1 indicating greater sensitivity toward the mitophagic removal of dysfunctional mitochondria [53]. HIIT-derived mechanical or metabolic stress appears to fine-tune this mitochondrial quality-control loop, with moderate cyclic shear increasing PINK1/Parkin turnover and mitochondrial fission/fusion cycling, concomitant with a reduction in the amount of ROS generated by mitochondria [54].

In an experimental model of type 2 diabetes, young male Wistar rats were subjected to an 8-week HIIT protocol consisting of 4–10 treadmill intervals at 80–100% of the individual maximal running velocity (Vmax). Compared with those in sedentary diabetic rats, significant increases in hippocampal lactate, MCT2, SIRT1, FOXO3, LC3, PINK1, and Parkin protein levels were detected, whereas a concomitant reduction in oxidative stress (↓ malondialdehyde (MDA), ↑ SOD/glutathione peroxidase (GPx)) and decreased amyloid-β and hyperphosphorylated tau accumulation were detected. The data suggested that the activation of lactate-driven PINK1/Parkin-dependent mitophagy in the hippocampus could protect the brain from the development of neurodegenerative conditions [55,56], which provides a partial rationale and mechanism explaining the reduced incidence of AD in highly active individuals, which is the subject of ongoing clinical trials such as sequential, multiple assignment, randomized trials (SMART) [57].

In mice, 5 weeks of voluntary wheel running (≈8.4 km/day) significantly increased the endothelial mitochondrial content in the descending thoracic aorta, as shown by en face porin (VDAC) immunostaining. Higher porin protein levels and increased mtDNA copy numbers were observed, indicating that exercise-induced laminar shear stress also enhances endothelial mitochondrial biogenesis in arteries exposed to sustained unidirectional flow [58]. Very recently, Hong et al. confirmed that laminar shear stress mimicking exercise conditions upregulated PINK1 expression and enhanced Parkin-dependent mitophagy in endothelial cells, thereby improving mitochondrial turnover, and reduced oxidative stress both in vitro in human primary ECs and in the aortic endothelium of exercising mice [53].

### 2.4. SASP as a Driver of Endothelial Dysfunction

Aged and senescent ECs remain metabolically active and adopt a SASP characterized by the chronic release of proinflammatory cytokines, particularly IL-6 and TNF-α [11], prothrombotic and profibrotic mediators such as PAI-1 [59], MMP-2, MMP-9, and stress–response factors such as GDF15 [60]. Together, these ‘SASP factors’ sustain low-grade vascular inflammation, drive extracellular matrix (ECM) remodelling and stiffening, and promote thrombofibrotic microenvironments, all of which accelerate vascular ageing and impair endothelial repair capacity [61,62].

IL-6 and TNF-α activate the NF-κB signalling pathway in neighbouring endothelial cells, upregulate the expression of ICAM-1 and VCAM-1, and reduce eNOS activity, which reduces NO bioavailability. This promotes inflammaging through increased leukocyte adhesion, oxidative stress, and a sustained inflammatory state within the vascular wall (reviewed by [63,64]). A study in young (8–10-week-old) and aged (58-week-old) mouse aortic ECs exposed to selective NOX1 inhibition with NOXA1 docking sequence inhibitory peptide (NOXA1ds) revealed that suppressed senescence was associated with reduced expression of DNA damage markers (SA-β-gal, γ-H2AX, p21/p16) and IL-6. In vivo delivery of NOXA1ds (20 mg/kg/day for 28 days) in 18-month-old mice reduced vascular NOX1/IL-6 protein expression and restored normal endothelial function, improving maximal acetylcholine-induced dilation [65].

Similarly, in a mouse model of ischaemic retinopathy, retinal neurons and microglia adopted a SASP senescent phenotype characterized by activation of the IRE1α ER stress pathway and significantly increased the expression of IL-6, PAI-1, IL-1β, and VEGF, which propagated paracrine senescence to ECs and drove pathological angiogenesis. Both genetic and pharmacological suppression of SASP signalling (via IRE1α inhibition or intravitreal metformin delivery, respectively) markedly reduce the senescence burden and pathological neovascularization in vivo [66].

At the cellular level, PAI-1 acts both as a marker and an effector of endothelial senescence, creating a prothrombotic and profibrotic microenvironment that stiffens the arterial wall and compromises vascular repair capacity [59]. This finding was demonstrated in a streptozotocin-induced diabetic mouse wound-healing model (male C57BL/6 mice), where lentiviral overexpression of PDK4 in full-thickness dorsal skin wounds markedly reduced fibroblast senescence, as evidenced by decreased SA-β-gal positivity and downregulation of p53, p21, p16^INK4a^, and the SASP factor PAI-1, concomitant with reduced IL-6 and MMP-3 expression. This effectively accelerated full-thickness wound closure and improved collagen deposition and angiogenesis [67]. SASP-associated MMPs further remodel the ECM by driving collagen cross-linking, elastin fragmentation, and medial calcification, which collectively lead to arterial stiffness and vascular senescence [62]. Simultaneously, they exhaust endothelial progenitor cell function and inhibit smooth muscle cell plasticity toward a contractile/reparative phenotype [11].

Specifically, circulating levels of GDF15, which were examined in a cohort of 4736 individuals, were shown to be correlated with greater arterial stiffness (by carotid–femoral pulse wave velocity) and age-related aortic stiffening and to be a strong predictor of all-cause mortality. These data strongly support its role as an integrative biomarker of vascular ageing and adverse clinical outcomes [68,69].

Collectively, these findings define the SASP as a paracrine amplifier of vascular ageing, wherein the persistent release of IL-6, TNF-α, PAI-1, MMPs, and GDF15 sustains endothelial inflammation, ECM remodelling, and the loss of regenerative potential. Targeting this network, either by senomorphic interventions or by direct modulation of NF-κB and PAI-1 signalling, represents a promising approach to attenuate vascular inflammaging and restore endothelial homeostasis.

## 3. Summary of Evidence and Hypotheses for Biomarker-Guided HIIT in the Optimization of Autophagy–Senescence Pathways for Vascular Health

HIIT has been widely studied for its vascular effects, including improvements in FMD, reductions in PWV and the modulation of redox status through increased antioxidant defences and reduced levels of oxidative stress markers such as MDA and oxidized LDL. HIIT may stimulate beneficial cellular adaptations, including autophagy and mitophagy, although direct measurements of these processes in human vascular tissue are scarce. Similarly, while HIIT has been associated with decreased markers of systemic inflammation, its influence on the SASP in the vascular endothelium remains largely undetermined.

Most HIIT studies report external training parameters such as intensity and duration, but few have investigated the induction of specific molecular responses such as autophagy or mitophagy, and even fewer have validated these findings with flux-based biomarkers in humans [35]. Interestingly, most recently, both Wistar rat and human skeletal muscle samples from four separate experiments assessing autophagy markers were examined, notably the LC3B-I and LC3B-II proteins. In rats, exercise induced a significant post-exercise increase in LC3B-I and a delayed increase in LC3B-II during recovery, without corresponding changes in mRNA levels. In contrast, human skeletal muscle exhibited a decrease in LC3B-II immediately after exercise regardless of intensity, which normalized after 3.5 h, but there was also no significant change in LC3B-I or MAP1LC3B mRNA. Notably, SQSTM1/p62 levels remained unchanged in both species. Ex vivo autophagy flux assays demonstrated that the reduction in LC3B-II in humans actually correlated with a modest-to-large increase in autophagic flux, lasting up to 24 h [70]. These findings highlight interspecies differences in autophagy marker dynamics and caution against the use of LC3B-II changes alone as proxies for autophagy activity in humans, hence providing support for the utilization of biomarker panels to assess overall impact on our biological systems.

However, no current protocol customizes interval prescriptions to generate a targeted shear stress stimulus (defined by the upstream signal for eNOS, AMPK, and SIRT1 activation), which is necessary for optimizing the autophagy–senescence balance. This review was designed to address a key translational gap by linking intracellular mechanisms, particularly autophagic flux and SASP regulation, to precision-tailored HIIT dosing strategies. Hence, our key hypothesis suggests that there is an individualized and dynamically changing HIIT “sweet spot” (work-bout intensity × bout duration × recovery) that maximizes autophagic flux and mitophagy in circulating vascular proxies while minimizing SASP, producing greater and longer improvements in FMD, PWV, and glycocalyx integrity and thereby providing the rationale for calibrating and personalizing HIIT. Further studies characterizing the expression and composition of endothelial progenitor cells, circulating extracellular vesicles, and vascular biopsy proxies may help validate this mechanistic window of optimization. Table 1 summarizes selected experimental models illustrating the interaction between exercise-induced shear stress and molecular pathways regulating endothelial autophagy and vascular function.

### 3.1. Defining and Estimating Shear Stress Dose in Humans; Physiological Relevance to HIIT

A central premise of vascular remodelling and endothelial adaptation to exercise is the magnitude and pattern of shear stress imposed on the arterial wall. Shear stress, which is defined as the tangential force per unit area exerted by flowing blood on the endothelial surface, serves as a mechanosensitive stimulus for key regulatory pathways, including eNOS, AMPK, and SIRT1, and downstream autophagy and mitochondrial biogenesis mediators [71]. However, most HIIT studies describe exercise in terms of external load or time without calibrating internal vascular mechanostimulation. This introduces a critical gap: molecular adaptations are likely dose-dependent on shear stress, not merely exercise intensity. In vivo estimation of shear stress during exercise remains challenging, particularly in clinical or laboratory settings. The most direct measure involves Doppler ultrasound to assess blood velocity and vessel diameter, allowing calculation of the wall shear rate (WSR) as

$$\mathrm{WSR}=\frac{4\times Q}{{\pi \times r}^{3}}$$

where Q is the blood flow, and r is the vessel radius. This approximation assumes laminar flow and is widely used in studies involving the brachial artery during rhythmic limb movement or cycling [72]. In applied settings where Doppler imaging is unavailable, surrogate markers of heart rate (HR), stroke volume (SV), and cardiac output (CO) can reliably be used to estimate the relative shear stimulus. Combining HR and estimated SV through impedance cardiography or wearable-derived algorithms is an acceptable substitute proxy for the measurement of systemic blood flow increases. Pulse pressure and vascular conductance should also provide appropriate and necessary insights into relative arterial loading and blood flow distribution, particularly in lower limb-focused protocols such as cycling or treadmill intervals.

### 3.2. Hypothetical Calibration for Translational Utility

To personalize HIIT for endothelial and mitochondrial benefits, it is essential to calibrate protocols against a target shear range, either individually or by phenotype clustering. This may involve pre-exercise vascular profiling via FMD to estimate responsiveness; adjusting interval intensity or duration to maintain shear within optimal adaptive windows (e.g., 5–20 dynes/cm^2^); and recording HR variability and recovery slopes to account for autonomic and vascular recovery dynamics between bouts. These parameters should be further refined by integrating wearable technologies, enabling real-time monitoring of heart rate, estimated VO_2_, and recovery kinetics to dynamically monitor vascular shear. In the realm of professional sports, where physiological performance is fine-tuned to the greatest degree, the ability to personalize cardiovascular and metabolic training stimuli is increasingly recognized as essential. Traditional exercise prescriptions often rely on percent-based heart rate or VO_2_max estimates. However, these proxies fail to account for interindividual variability in vascular responsiveness, particularly at the endothelial level, where shear stress is a primary driver of nitric oxide production, endothelial repair, and vascular remodelling.

Understanding the concept of a “shear dose” (the cumulative shear stimulus exerted on the vascular endothelium during a training session) represents the next level of personalized training. In professional athletes, shear dose estimation can be monitored via Doppler ultrasound to quantify blood velocity and vessel diameter (τ = 4μQ/πr^3^), [73], or more practically through heart rate × stroke volume approximations. These surrogates should enable labs and performance centres to calibrate training loads not only for performance output but also for vascular adaptation efficiency, particularly in recovery periods and during HIIT cycles. This allows for periodized vascular conditioning, minimizes endothelial fatigue, optimizes mitochondrial biogenesis via AMPK/SIRT1/PGC-1α, and prevents maladaptive arterial remodelling. This is crucial for endurance athletes, cyclists, and high-load intermittent sports (e.g., football, rugby), where the balance between training stress and vascular resilience determines longevity and injury risk. So how might this look if we attempt to combine it with a biomarker stratifying platform?

### 3.3. Incorporation and Design of the Biopharmacological Biomarker Panels

To assess the mechanistic impact of HIIT on vascular health and ageing, particularly regarding autophagy and senescence, it is essential to implement a validated, multitiered biomarker panel. This panel should capture both acute signalling changes and longer-term adaptations in terms of cellular health, mitochondrial quality, and inflammation resolution. Biomarkers measured in blood, specifically from PBMCs and circulating EVs, offer a minimally invasive window into systemic responses to exercise and vascular stress. Autophagy is a dynamic process that requires the measurement of flux (i.e., the rate of autophagosome formation and clearance), not just static protein levels. In human studies, PBMCs have been demonstrated to be a feasible tissue source for detecting autophagy flux, particularly LC3B-II accumulation in the presence and absence of lysosomal inhibitors such as bafilomycin A1. This approach enables, for example, differentiation between increased degradation and increased synthesis of autophagosomes [74]. Additionally, circulating EVs can serve as reservoirs for the key autophagy-related and mitophagy-related proteins PINK1 and Parkin, which are released from stressed tissues such as muscle and endothelium. The quantification of mitochondrial DNA copy number (mtDNAcn) in EVs or plasma should also reflect mitophagic activity and mitochondrial health and senescence-associated traits in addition to age-related and specific SASP-related traits ([75]; reviewed by [76]).

As described above, chronic HIIT or maladapted protocols accelerate cellular senescence or exacerbate the SASP, especially in endothelial or immune cells. Measuring p16^INK4a^, among other SASP markers in sorted CD3^+^ T cells, has emerged as a robust ageing biomarker and is sensitive to both training status (reduced in exercising individuals) and chronic inflammation [77]. Other circulating SASP markers that should be considered in a biomarker panel include IL-6, TNF-α, PAI-1, osteopontin and GDF15, which are utilized as markers of cellular stress and mitochondrial distress and are often upregulated with ageing and cardiometabolic disease [78].

**Figure 4:**
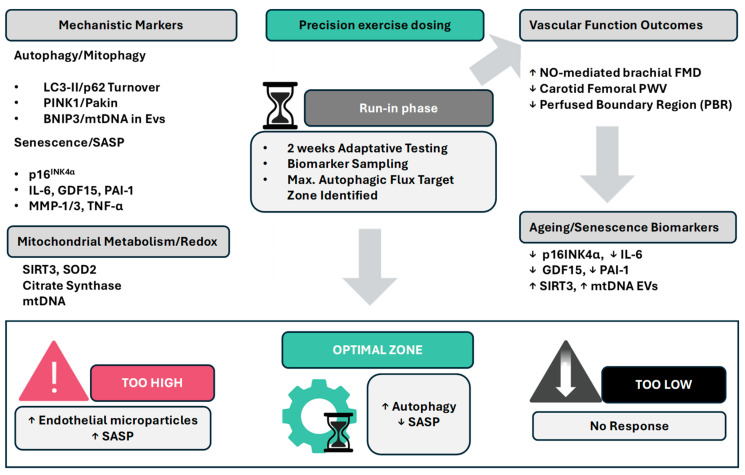
Precision exercise dosing algorithm linking shear stress to endothelial autophagic flux, SASP suppression, and vascular outcomes. The schematic illustrates a mechanistically guided framework for individualized exercise prescription based on endothelial autophagy and senescence signalling. During an initial run-in phase, cardiopulmonary testing is used to determine individualized exercise workloads that generate specific arterial shear profiles. Shear stress can be estimated directly using Doppler-derived shear rate measurements or indirectly through wearable-based proxies integrating heart rate and stroke volume. Acute biomarker sampling following exercise enables identification of the shear range associated with maximal autophagic flux. Panels assessing autophagy and mitophagy markers (LC3-II, p62/SQSTM1, PINK1/Parkin, BNIP3/NIX) together with senescence and SASP indicators (p16^INK4a^, IL-6, TNF-α, GDF15, PAI-1, MMPs) and mitochondrial/redox markers (SIRT3, SOD2, NRF2 targets, mitochondrial DNA) allow evaluation of endothelial adaptive responses to exercise-derived shear stress (p16^INK4a^ in T cells, IL-6, TNF-α, GDF15, PAI-1, MMP-1/3, and osteopontin) can identify the inflammatory and profibrotic response to shear stress, whereas redox and mitochondrial adaptations can be indexed by SIRT3, SOD2, NRF2 target gene expression, citrate synthase activity, and mitochondrial DNA copy number. (IL-6—interleukin-6, TNF-α—tumour necrosis factor-alpha, GDF15—growth differentiation factor 15, PAI-1—plasminogen activator inhibitor-1, MMP-1/3—matrix metalloproteinase 1/3, LC3-II—light chain 3, lipidated form (form II), p62/SQSTM1—sequestosome 1, PINK1/Parkin—PTEN-induced kinase 1/Parkin (PARK2), SASP—senescence-associated secretory phenotype, SIRT3—sirtuin 3, SOD2—superoxide dismutase 2, NRF2—nuclear factor erythroid-2-related factor 2, ↑ up arrow means increase, ↓ down arrow means decrease). Created in BioRender. Tero-Vescan, A. (2026) https://BioRender.com/yumf3xr (accessed on 5 March 2026).

Together, these markers enable mapping of the inflammatory milieu, oxidative stress state, and regenerative balance post HIIT. Accurate biomarker evaluation must also account for temporal dynamics. Two critical windows are defined: The acute response window (3–4 h post HIIT): This window is optimal for assessing transient molecular signals (e.g., increased autophagic flux, IL-6 spikes, and EV cargo changes). Measurements during this window should reflect immediate endothelial and immune stress responses. Chronic adaptation (4–8 weeks of repeated HIIT): This window captures long-term vascular and metabolic remodelling, including restored mitochondrial biogenesis, reduced SASP marker expression, and improved vascular function (e.g., FMD, PWV). Paired comparisons of baseline and endpoint samples during this period will also enable assessment of net beneficial or maladaptive outcomes. This dual-window strategy ultimately ensures better mechanistic resolution of both signalling onset and phenotypic plasticity in response to HIIT. Figure 4 shows a brief description of what such a protocol and algorithm would look like in practice.

The algorithm defines an “optimal shear zone”, characterized by increased autophagic and mitophagic flux with concomitant suppression of SASP mediators. Exercise intensities below this zone fail to trigger sufficient autophagy and produce minimal vascular benefit (“too low”). Conversely, intensities exceeding this zone are expected to promote endothelial stress, as reflected by increased endothelial microparticle release and reactivation of SASP signalling (“too high”). Following run-in calibration, participants would enter a randomized crossover design, comparing conventional HIIT protocols with shear-calibrated HIIT, in which interval intensity, cadence, and recovery duration are dynamically adjusted to maintain shear stress within the identified optimal range. Primary vascular outcomes will include conduit artery flow-mediated dilation (FMD; NO-dependent endothelial function) and carotid–femoral pulse wave velocity (PWV; arterial stiffness), complemented by microvascular and glycocalyx assessments such as sublingual perfused boundary region (PBR), retinal microvascular indices, and skin laser Doppler flowmetry. Positive responders will then be defined by a prespecified increase in autophagic flux, and mediation analyses will be performed to test whether improvements in vascular function can be statistically explained by changes in autophagy and SASP suppression. Basic algorithmic equations for the measurement of shear and biomarker integration are shown below and serve as hypothesis-derived examples only.

A composite response index integrating autophagy- and mitophagy-related markers (ΔLC3-II, ΔPINK1, and ΔSIRT3) and subtracting inflammatory and stress-related markers (ΔIL-6 and ΔGDF15), which are expressed as z scores normalized to baseline values, can be used to quantify biological responses to shear stress. The optimal shear dose (shear dose) in this case was defined as the value maximizing the response index within a physiological range.

$$Response\;Index\left(Shear\;Dose\right)=\left[w_{1}z\left(∆LC3-II\right)+w_{2}z\left(∆PINK1\right)+w_{3}z\left(∆SIRT3\right)\right]-\left\lfloor v_{1}z\left(∆IL-6\right)+v_{2}z(∆GDF15)\right\rfloor$$

In the equation, the individualized shear dose (shear dose*) was calculated as the time-integrated shear rate derived from Doppler-based blood velocity and vessel diameter measurements. The individualized shear dose was defined as the shear stress value that maximized the composite response index within a physiologically relevant range.

$${Shear\;Dose}^{*}={arg}_{ShearDose\in [low,high]}max\;Response\;Index\;(Shear\;Dose)$$

The shear dose was operationally defined as the time integral of the instantaneous shear rate, derived from Doppler-measured mean blood velocity and vessel diameter.

$$Shear\;Dose=\int_{0}^{T}\dot{\gamma }\left(t\right)\;dt\;with\;\dot{\gamma }\left(t\right)=\frac{4V_{mean}(t)}{D(t)}$$

where ∫_0_ᵀ … dt represents the time integral, representing the accumulation of shear stress over the total duration of exposure; t is the total duration of the exercise or measurement period; $\dot{\gamma }$(t) (instantaneous shear rate) is the instantaneous rate of shear experienced by the endothelial surface at time t; V_mean_(t) is the mean blood flow velocity measured by Doppler ultrasound at time t; D(t) is the instantaneous vessel diameter at time t; and 4 is a scaling factor derived from the assumption of laminar parabolic flow within the vessel and dt is an infinitesimal time increment, allowing the integration of the shear rate over time.

The mechanostimulus of each exercise session is quantified as the time-integrated conduit–artery shear rate, estimated from Doppler-derived mean blood velocity (V_mean_) and arterial diameter (D). To link the shear dose to biology, we defined a composite response index that rewards the activation of endothelial quality control pathways while penalizing inflammatory stress signalling. Specifically, LC3-II is used as a readout of autophagosome formation (interpreted in the context of flux assays where available), PINK1 indexes mitophagy priming/mitochondrial damage sensing, and SIRT3 reflects mitochondrial redox resilience and oxidative phosphorylation homeostasis. In contrast, IL-6 and GDF15 are included as adverse response terms capturing acute inflammatory activation and mitochondrial/stromal stress signalling that can accompany excessive or poorly tolerated shear exposure. All biomarker changes are z scores normalized to place heterogeneous assays on a common scale. The optimal individualized exercise dose is defined as the shear dose that maximizes this composite index within a prespecified safe range, operationalizing the concept that beneficial vascular adaptation occurs within an “optimal basin” where autophagy/mitophagy signalling increases while proinflammatory stress responses remain constrained.

Within this composite biological response score, we integrated adaptive (autophagy/mitophagy) and maladaptive (inflammatory stress) signalling induced by exercise-derived shear stress. Definitions and terms: ShearDose: The time-integrated endothelial shear stimulus over an exercise session, calculated as the integral of the shear rate over time. ShearDose*: Individualized optimal shear dose that maximizes the response index within predefined safety limits. $\dot{\gamma }$(t) (shear rate): Instantaneous conduit artery shear rate at time t, expressed in s^−1^. V_mean_(t): Time-varying mean blood flow velocity measured by Doppler ultrasound (m·s^−1^). D(t): Time-varying arterial internal diameter measured by ultrasound (m). Δ: Acute change in a biomarker from pre-exercise baseline (e.g., 3–4 h post-exercise minus baseline). z(⋅): Z score normalization of biomarker change, defined as the change divided by the standard deviation of that biomarker within the study cohort. w1, w2, w3: Positive weighting coefficients assigned to adaptive biological responses reflecting endothelial quality control and mitochondrial health. v1, v2: Positive weighting coefficients assigned to maladaptive inflammatory or stress responses.

### 3.4. Conclusions: Implications for Clinical and Translational Research

A “calibrated shear” approach bridges the gap between exercise physiology and molecular signalling, offering a pathway for precision exercise prescription. Importantly, defining a shear dose threshold linked to autophagy induction and senescence suppression may unlock new therapeutic windows in ageing populations and those with early vascular dysfunction or preclinical cardiometabolic disease.

Accumulating evidence now supports endothelial autophagic flux and SASP signalling as central regulators of vascular ageing and cardiometabolic risk. This review integrates mechanistic insights from shear-sensitive signalling pathways with emerging biomarker methodologies to propose a unifying framework in which hemodynamic forces, mitochondrial quality control, and inflammatory restraint converge to determine endothelial fate. By highlighting AMPK–SIRT1–PGC-1α/NRF2 as a novel axis, as well as PINK1–Parkin–dependent mitophagy and ULK1–LC3 autophagic throughput as shear-responsive nodes, we emphasize that endothelial adaptation to exercise is governed not by exercise intensity per se but by the biological response elicited within a defined mechanobiological window. Importantly, failure to achieve sufficient autophagic flux or exposure to excessive or disturbed shear may instead amplify SASP signalling, endothelial microparticle release, and vascular dysfunction, providing critical evidence of the need to move beyond “one-size-fits-all” exercise prescriptions.

From a translational perspective, the proposed precision exercise dosing approach offers a path toward individualized vascular therapy. By integrating shear-dose calibration with dynamic biomarker readouts of autophagy mitochondrial resilience and inflammatory stress, this approach enables identification of an optimal adaptive zone in which vascular benefits are maximized while maladaptive stress responses are minimized. This framework has immediate implications for populations at heightened cardiovascular risk, including individuals with diabetes, metabolic syndrome, and advanced vascular ageing, where indiscriminate high-intensity training may be ineffective or harmful. Future interventional trials incorporating shear-calibrated exercise and biomarker-guided stratification will be essential to validate this model, but the conceptual shift is clear: exercise should be considered a precision mechanotherapeutic intervention, with endothelial autophagic flux and SASP suppression serving as measurable, actionable targets to preserve vascular health across the lifespan.

## Figures and Tables

**Table 1:** Selected experimental models illustrating the interaction between exercise-induced shear stress and molecular pathways regulating endothelial autophagy and vascular function.

Model/Population	Exercise or Shear Stimulus	Main Molecular Pathways	Key Biomarkers/Molecular Findings	Vascular Outcome	Ref.
SHR model	High-intensity treadmill training, 12 weeks (70–80% VO_2_max)	AMPKα–SIRT1 signalling	↑ eNOS Ser1177 phosphorylation, ↓ NOX4, ↓ DHE, ↓ 8-OHdG, ↓ NLRP3 inflammasome	Improved endothelium-dependent aortic relaxation and reduced endothelial oxidative stress	[31]
Mouse model	Voluntary wheel running (≈8.4 km/day) for 5 weeks	Shear stress-induced mitochondrial biogenesis	↑ VDAC/porin, ↑ mtDNA copy number	Increased endothelial mitochondrial content in the thoracic aorta	[58]
Aged mouse model	Chronic exercise training	PPARγ–FUNDC1 mitophagy pathway	Increased mitophagic flux and reduced senescence markers	Improved microvascular function	[37]
Wistar rats and human skeletal muscle	Acute exercise protocols (various intensities)	LC3B-I, LC3B-II, SQSTM1/p62	Rats: ↑ LC3B-I post exercise and delayed ↑ LC3B-II during recovery; humans: ↓ LC3B-II immediately after exercise with normalization at 3.5 h; p62 unchanged	Increased autophagic flux in human skeletal muscle despite reduced LC3B-II levels	[70]
Moderately trained healthy men (n = 40)	8-week aerobic training (3 sessions/week); comparison of long slow distance (70% HRmax), lactate threshold (85% HRmax), and HIIT protocols (15/15 intervals or 4 × 4 min at 90–95% HRmax)	Cardiovascular adaptations associated with high-intensity interval exercise	↑ VO_2_max (5.5–7.2% in HIIT groups); ↑ stroke volume (~10%); improvements in lactate threshold and running economy	Improved cardiorespiratory fitness and cardiovascular efficiency	[5]
Moderately trained young men (≈25 yrs)	8-week cycling training with intermittent 30 s sprints	AMPK–ULK1 autophagy signalling; mitophagy regulation	↑ AMPK Thr172, ↑ ULK1 Ser317 phosphorylation; ↑ LC3-I/II and BNIP3 during recovery; chronic training ↑ OXPHOS complex I, Parkin, BNIP3	Enhanced skeletal muscle autophagy and mitochondrial quality control	[35]

Abbreviations: AMPK, AMP-activated protein kinase; SIRT1, sirtuin 1; eNOS, endothelial nitric oxide synthase; NOX4, NADPH oxidase 4; DHE, dihydroethidium; 8-OHdG, 8-hydroxy-2′-deoxyguanosine; NLRP3 inflammasome, NOD-like receptor family pyrin domain containing 3 inflammasome; VDAC, voltage-dependent anion channel; mtDNA, mitochondrial DNA; PPARγ, peroxisome proliferator-activated receptor gamma; FUNDC1, FUN14 domain-containing protein 1; LC3B-I and LC3B-II, microtubule-associated protein 1 light chain 3B forms I and II; SQSTM1/p62, sequestosome 1; OXPHOS, oxidative phosphorylation; BNIP3, BCL2/adenovirus E1B 19 kDa interacting protein 3, ↑ up arrow means increase, ↓ down arrow means decrease.

## Data Availability

No new data were created or analyzed in this study. Data sharing is not applicable to this article.

## Abbreviations

The following abbreviations are used in this manuscript:

ACE	angiotensin-converting enzyme
AD	Alzheimer’s disease
AMP/ATP	adenosine monophosphate/triphosphate
AMPK	adenosine monophosphate-activated protein kinase
ATG13	autophagy-related protein 13
BH_2_/BH_4_	di/tetrahydrobiopterin
CAECs	coronary artery endothelial cells
CO	cardiac output
Cat	catalase
EC	endothelial cell
EMP	endothelial microparticle
eNOS	endothelial nitric oxide synthase
EVs	Extracellular vesicles
FIP200	FAK family-interacting protein of 200 kDa
FMD	flow-mediated dilation
FOXO1	forkhead box O transcription factor 1
GDF15	growth differentiation factor 15
GLUT4	glucose transporter 4
GPx	glutathione peroxidase
HAECs	human aortic endothelial cells
HR	heart rate
HIIT	high-intensity interval training
HO-1	heme oxygenase-1
HUVECs	human endothelial cells
IL-6	interleukin-6
KLF2	Krüppel-like factor 2
LC3	microtubule-associated protein 1A/1B-light chain 3
LC3-II	light chain 3, lipidated form (form II)
LSS	low shear stress
MDA	malondialdehyde
MICT	moderate-intensity continuous training
MMP	matrix metalloproteinase
mTORC1	mechanistic target of rapamycin complex 1
NAD^+^/NADH+H^+^	nicotinamide adenine dinucleotide (oxidized/reduced)
NF-κB	nuclear factor kappa-light-chain-enhancer of activated b cells
NO	nitric oxide
NOX2	NADPH oxidase 2
NRF2	nuclear factor erythroid-2-related factor 2
OMM	outer mitochondrial membrane
OXPHOS	oxidative phosphorylation
P	phosphate group
p62/SQSTM1	sequestosome 1
PAI-1	plasminogen activator inhibitor-1
PBMCs	peripheral blood mononuclear cells
PGC-1α	peroxisome proliferator-activated receptor-gamma coactivator-1α
PINK1	PTEN-induced kinase 1
PINK1/Parkin	PTEN-induced kinase 1/Parkin (PARK2)
PPAR-γ	peroxisome proliferator-activated receptor gamma
PWV	pulse wave velocity
RhoA	Ras homologue family member A
ROCK	Rho-associated coiled-coil-containing protein kinase
ROS	reactive oxygen species
SASP	senescence-associated secretory phenotype
SHRs	spontaneously hypertensive rats
SIRT1	sirtuin 1
SOD2	superoxide dismutase 2
SV	stroke volume
TNF-α	tumour necrosis factor-alpha
UBQ	ubiquitin,
UCP2	uncoupling protein 2
ULK1	unc-51 like autophagy-activating kinase 1
VEGF	vascular endothelial growth factor
VPS34	vacuolar protein sorting 34
WSR	wall shear rate
